# *Passiflora edulis* seed oil from west Cameroon: Chemical characterization and assessment of its hypolipidemic effect in high‐fat diet–induced rats

**DOI:** 10.1002/fsn3.1234

**Published:** 2019-10-22

**Authors:** Prosper Ngakou Takam, Fabrice Tonfack Djikeng, Dieudonné Kuate, Anne Pascale Nouemsi Kengne, Hermine Doungué Tsafack, Inelle Makamwé, Julius Enyong Oben

**Affiliations:** ^1^ Laboratory of Biochemistry, Medicinals Plants, Food Sciences and Nutrition (LABPMAN) Department of Biochemistry University of Dschang Dschang Cameroon; ^2^ Laboratory of Nutrition and Nutritional Biochemistry Department of Biochemistry University of Yaounde I Yaounde Cameroon; ^3^ School of Agriculture and Natural Resources Catholic University Institute of Buea Buea Cameroon

**Keywords:** fatty acid composition, hypolipidemic, *Passiflora edulis*, rats, seed oil

## Abstract

In this study, the in vivo hypolipidemic effect of west Cameroonian *Passiflora edulis* variety seed oil (PE) was assessed in female and male *Wistar* rats. The chemical properties of the oil were evaluated through the determination of the peroxide, iodine, and thiobarbituric acid values, as well as its fatty acid composition using gas chromatography. Results showed that the oil extraction yield was 19.90% and its quality indexes were as follows: peroxide value = 2.10 ± 0.20 meq O_2_/kg; thiobarbituric acid value = 0.25 ± 0.00 ppm; and iodine value = 97.40 ± 0.45 g I_2_/100 g. Its fatty acid composition showed that it contains about 84.88% of unsaturated fatty acid, linoleic acid being the most represented (68.39%), followed by oleic acid (14.31%). The administration of this oil resulted in a significant reduction (*p* < .05) in the level of triglycerides, total cholesterol, and low‐density lipoprotein‐cholesterol in rats. The PE groups showed a significant increase (*p* < .05) in high‐density lipoprotein‐cholesterol compared with untreated male rats. A similar trend was observed with female rats for triglycerides, but lowest values were observed with olive oil at 1 ml. This study suggests that *P. edulis* seed oil is rich in linoleic acid, which might be responsible for its hypolipidemic effect comparable to that of olive oil.

## 1 INTRODUCTION

Cardiovascular disease (CVD) remains one of the most common public health problems of global significance. In the last decades, hyperlipidemia, also known as dyslipidemia, has largely been documented as a risk factor for CVD (Kulczyński, Gramza‐Michałowska, Kobus‐Cisowska, & Kmiecik, 2017). Hyperlipidemia is defined as a chronic metabolic disease characterized by abnormal concentration of blood lipid levels (high levels of total cholesterol, total triglycerides, and LDL‐c and low level of HDL‐c) leading to various ranges of chronic metabolic diseases (Ding, Pu, & Kan, 2017; Soedamah‐Muthu et al., 2003).

Current treatments of dyslipidemia include hypolipidemic agents such as niacin, statins, and fibrates, which represent the most common method of controlling blood lipid profile (Sashidhara, Kumar, Kumar, Srivastava, & Puri, 2010). However, these pharmacological methods are associated with high cost of procurement, several adverse effects, and low efficacy. Previous studies have indicated that diet and particularly dietary lipids play important roles in controlling blood lipid levels. Polyunsaturated fatty acid (PUFA)—rich oils, because of their LDL‐c‐ and triglyceride‐lowering properties, have received considerable attention in prevention and treatment of hyperlipidemia and atherosclerosis. Omega‐3 and omega‐6 fatty acids, most documented, have been shown to increase blood HDL‐c (generally described as “good” cholesterol) because of their implication in transporting cholesterol from the blood to the liver (Cantwell, 2000; Kris‐Etherton & Yu, 1997; Prabhavathi Devi et al., 2018).

*Pa* *ssiflora edulis*, also known as passion fruit, is widely consumed worldwide and represents a good source of PUFAs and exhibits many pharmacological properties. Seeds from this fruit generally contain oil ranging from 18.5% to 29.4%. Regarding its composition, this oil generally exhibits large amounts of linoleic acid (55%–66%), oleic acid (18%–20%), palmitic acid (10%–14%), and linolenic acid (~1%). However, this oil content and chemical composition are slightly influenced by clinical factors, environment, production zone, harvest period, growing condition, and variety (Nyanzi, Carstensen, & Schwack, 2005; Tenyang et al., 2017).

This study was designed to evaluate *P. edulis* seed oil from west Cameroon through assessment of its fatty acid profile, chemical properties, and hypolipidemic effect.

## 2 MATERIAL AND METHODS

### 2.1 Plant material

*Passiflora edulis* was identified at the National Herbarium of Cameroon under the following code: 65104/HNC. *Passiflora edulis* fruits were purchased in a local market of Dschang, Cameroon, between August and September 2016. After sorting and cleaning, fruits were manually opened and seeds extracted. These seeds were washed using water and dried for two days at 45°C using a ventilated air‐drying oven.

### 2.2 Extraction

These seeds (dried) were ground in a Binatone brand household blender to obtain a powder prior extraction. Oil was extracted from the powder by hexane maceration, according to the Womeni et al. (2016) protocol. In principle, the power (250 g) from *P. edulis* seeds was macerated in 800 ml hexane at room temperature for 24 hr with constant shaking. The mixture was filtered using Whatman paper No. 1, the filtrate was concentrated on a rotatory evaporator at 40°C, and the residue was dried in a ventilated air‐drying oven. The oil obtained was stored in a refrigerator at 4°C for further analyses.

### 2.3 Fatty acid profile using gas chromatography

Fatty acid methyl esters (FAMEs) of *P. edulis* seed oil were prepared by transesterification using 2% sulfuric acid in methanol (Christie, 1993). Two drops of this oil were mixed with 50 ml methanolic solution of sulfuric acid 2%; afterward, the mixture was heated under reflux at 95–100°C for 4 hr. After cooling, ethyl acetate was added in the flask to scavenge fatty acid methyl esters. The mixture was then transferred to a funnel and washed thrice with water. The organic phase was dried with purified anhydrous sulfate and the organic solvent eliminated by evaporation under pressure on a rotatory evaporator. Fatty acid methyl esters were collected by the addition of 1 ml of chloroform and analyzed in a gas chromatograph coupled to flame ionization detector (GC/FID).

The GC/FID analyses were performed with an Agilent (Agilent Technologies) 7890A series gas chromatograph equipped with FID detector using a DB‐225 capillary column (30 m × 0.25 mm, 0.25 µm of film thickness). The column temperature was initially maintained at 160°C for 2 min, increased to 220°C at 5°C/min, and maintained for 10 min at 220°C. The gas used was nitrogen at a flow rate of 1.5 ml/min. The injector and detector temperatures were maintained at 230 and 250°C, respectively, with a split ratio of 50:1. Identification of fatty acids was based on comparison of their retention time with that of standard reference fatty acid methyl esters (C_4_–C_20_) performed under the same conditions. Quantification of the FAME composition was done considering the relative areas of peaks, expressed as the relative percentage of the individual area with regard to total area of compounds in the chromatogram.

### 2.4 Chemical characterization of *Passiflora edulis* seed oil

The peroxide value of *P. edulis* seed oil was obtained following the spectrophotometric IDF standard method 74A: 1991 (IDF, 1991). The iodine value essay was carried out according to the AOCS official method CD 1–25 (AOCA, 2003). The thiobarbituric acid value was evaluated as described by Draper and Hadley (1990).

### 2.5 Animal model and experimental design

Seventy‐two *Albinos* rats of *Wistar* strain were used in this study. They were bred at the animal house of the Department of Biochemistry of the University of Dschang, Cameroon. They were 90 days old, weighing between 150 and 250 g. All rats (36 males and 36 females) were individually housed in a cage under 12‐hr light–dark cycle throughout the experimental period. Animals were randomly assigned to six groups of twelve rats (six males and six females) and were fed with a standard diet and water ad libitum for 1 week to stabilize their metabolism prior to experimentation. After the acclimatization period, five of the six groups were fed with high‐fat diet for one month (induction period), while the last one was fed with a standard diet. Subsequently, while maintaining a high‐fat diet, rats were orally supplemented with *P. edulis* seed oil and olive oil (used as a control) once a day for 30 days, using a gastroesophageal tube. All experiments were carried out according to the regulations and ethical approval of the Experimental Animal Welfare and Ethic Committee of the institution. Briefly, they were distributed as follows:

- Group 1: high‐fat diet + 1 ml/kg of body weight/day of distilled water;
- Group 2: high‐fat diet + 1 ml/kg of body weight/day of *P. edulis* seed oil;
- Group 3: high‐fat diet + 2 ml/kg of body weight/day of *P. edulis* seed oil;
- Group 4: high‐fat diet + 1 ml/kg of body weight/day of olive oil;
- Group 5: high‐fat diet + 2 ml/kg of body weight/day of olive oil;
- Group 6: standard diet + 1 ml/kg of body weight/day of distilled water.

The rats had free access to food and water. Their food intake and body weight were measured daily throughout the experimental period (28 days). At the end of the study, daily food intake, body weight gain, food efficiency ratio (expressed as a percentage of body weight gain/total food intake), and growth rate were calculated.

### 2.6 Lard preparation and diet composition

Lard purchased from the local market was melted in combination with unrefined palm oil in the ratio 5:3 (w/v). Palm oil was heated until loss of red color before introduction of lard. The mixture was then heated until complete solubilization of lard and cooled at room temperature, followed by filtration. A filtrate and a black solid residue were obtained from the melted lard. This filtrate was used in the composition of the high‐fat diet as follows: The filtrate was mixed with a standard diet (with 1% cholesterol and 0.5% cholic acid) in the ratio 1:3 as described in the Sayin et al. (2016) protocol. The composition of each experimental diet is shown in Table 1.

**Table 1:** Composition of diets

Ingredients (for 1,000 g)	Standard diet	Hyperlipidemic diet
Corn flour	680	500
Soy flour	200	150
Fish meal	100	75
Bonemeal	10	6.5
Cooking salt	8	6
Cholic acid	0	1
Cholesterol	0	10
Refined palm oil	1	0.75
Lard	0	250
Vitamins and mineral mix	1	0.75

### 2.7 Serum collection and lipid profile assessment

Twelve hours of fasting after the last gastroesophageal administrations, rats were slightly anesthetized and blood samples were collected from the abdominal aorta and placed into laboratory test tubes containing without anticlotting substance. Animals were promptly sacrificed by cervical vertebra dislocation, and vital organs (liver, kidney, and heart) were isolated. After 30 min at room temperature, clotting blood samples were centrifuged at 3000*g* for 15 min. The supernatants (sera) were collected using micropipettes and stored in Eppendorf tubes at −20°C for biochemical analyses.

Total cholesterol (TC), triglycerides (TG), and high‐density lipoprotein cholesterol (HDL‐c) were assessed by the photometric method using Chronolab commercial kits. Very low‐density lipoprotein cholesterol (VLDL‐c) was estimated from TG ([VLDL‐c] = [TG]/5), and low‐density lipoprotein cholesterol (LDL‐c) was estimated using the Friedewald equation ([LDL‐c] = [TC] − ([HDL‐c] + estimated [VLDL‐c])). These concentrations were expressed in mg/dl. Atherogenic index (AI) was calculated as AI = total blood cholesterol/total blood HDL‐c.

### 2.8 Statistical analyses

Statistical analyses were performed using IBM SPSS Statistics for Windows (version 20). One‐way analysis of variance (ANOVA) with post hoc Tukey test was performed, followed by Kruskal–Wallis test to compare the means of the six groups. Homogeneity of variances was tested using Levene's test. Correlations were done using the Pearson correlation significance test. The results were expressed as means ± standard deviations (*SD*), and significance was considered at *p* < .05.

## 3 RESULTS

### 3.1 Physical characteristics and extraction yield of *Passiflora edulis* oil

The oil obtained after extraction was yellow gold in color, liquid at room temperature, with an odor characterizing the passion fruit. The oil yield was found to be 19.90%.

### 3.2 Fatty acid composition of *Passiflora edulis* seed oil

**Figure 1:**
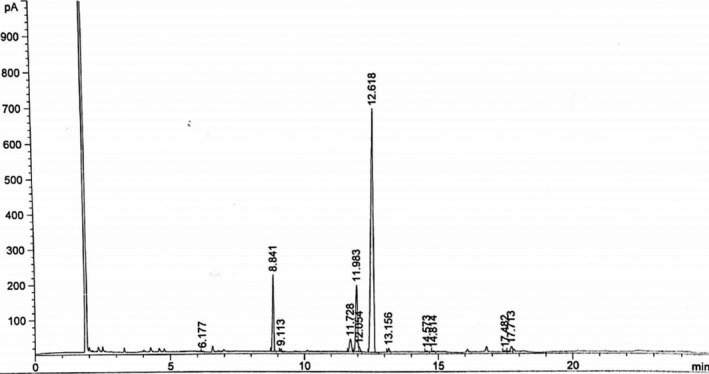
Characteristic gas chromatography–flame ionization detector chromatogram of the fatty acids from Passiflora edulis seed oil (main fatty acid peaks at retention times: 6.177 min, myristic acid C14:0; 8.841 min, palmitic acid C16:0; 9.113 min, palmitoleic acid C16:1; 11.728 min, stearic acid C18:0; 11.983 min, oleic acid C18:1; 12.618 min, linoleic acid C18:2; 13.156 min, ɑ‐linolenic acid C18:3; 14.573 min, arachidic acid C20:0; 14.814 min, gadoleic acid C20:1; 17.482 min, behenic acid C22:0; 17.713 min, cetoleic acid C22:1)

The fatty acid composition of *P. edulis* oil is presented in Table 2 and Figure 1. Five saturated fatty acids and six unsaturated fatty acids were detected. Linoleic acid (C18:2), oleic acid (C18:1), palmitic acid (C16:0), and stearic acid (C18:0) were the major fatty acids detected. Linoleic acid (C18:2) was the most represented fatty acid, with a percentage of abundance of 68.39%. It was followed by oleic acid (14.31%), palmitic acid (11.72%), and stearic acid (2.84%). The other fatty acids were present in traces (<1%).

**Table 2:** Fatty acid composition of *Passiflora edulis* seed oil

Code	Fatty acid name	Quantity (%)
C14:0	Myristic acid	0.10
C16:0	Palmitic acid	11.72
C16:1	Palmitoleic acid	0.34
C18:0	Stearic acid	2.84
C18:1	Oleic acid	14.31
C18:2	Linoleic acid	68.39
C18:3	ɑ‐Linolenic acid	0.54
C20:0	Arachidic acid	0.16
C20:1	Gadoleic acid	0.15
C22:0	Behenic acid	0.24
C22:1	Cetoleic acid	1.15

### 3.3 Chemical characterization of *Passiflora edulis* seed oil

The results of the chemical characterization of *P. edulis* oil are presented in Table 3. The iodine value was found to be 97.4 ± 0.45 g I_2_/100 g, the peroxide value was 2.10 ± 0.20 meq O_2_/kg, and the thiobarbituric acid value was 0.25 ± 0.00 ppm.

**Table 3:** Quality characteristics of passion fruit (Passiflora) seed oil

Oil characteristics	Values	Literature values (Cantwell, 2000)			
		Cotton seed oil	Corn oil	Palm oil	Palm kernel oil
Oil yield (%)	19.90				
Iodine value (g I_2_/100 g)	97.40 ± 0.45	100–123	103–135	50.0–55.0	14.1–21.0
Peroxide value (meq O_2_/kg)	2.10 ± 0.20				
Thiobarbituric acid value (ppm)	0.25 ± 0.00				
Unsaturated fatty acids (%)	84.88				
Saturated fatty acids (%)	15.06				
SFA/UFA	1/5.64				
Oleic/linoleic acid ratio	0.21				

Abbreviations: ppm, parts per million; SFA, saturated fatty acid; UFA, unsaturated fatty acid.

### 3.4 Effect of *Passiflora edulis* on food intake and growth

The effect of passion fruit seed oil on the growth characteristics of rats is shown in Table 4. Daily food intake did not differ (*p* < .05) among the treatment groups but was significantly different (*p* < .05) from that of the group receiving the standard diet in both sexes. At the end of the experiment, the body weight gain was higher in the high‐fat diet groups compared with the standard diet group, with the exception of the group treated with 1 ml/kg of body weight of olive oil which was lower than that of all the other groups in male rats, whereas in female rats, the lowest value was obtained with the group treated with 2 ml/kg of body weight of *P. edulis* seed oil.

**Table 4:** Body weight and food intake of control and experimental groups in male and female rats

	PC	PO 1 ml	PO 2 ml	OL 1 ml	OL 2 ml	NC
Male rats						
Initial body weight (g)	282.00 ± 9.42^ab^	293.00 ± 48.25^ab^	246.00 ± 17.28^ab^	241.33 ± 34.18^a^	305.67 ± 41.35^ab^	313.00 ± 18.40^b^
Final body weight (g)	319.00 ± 4.32^ac^	325.33 ± 50.39^ad^	280.33 ± 21.64^ab^	262.33 ± 37.14^a^	346.67 ± 43.79^bcd^	338.67 ± 17.93^bcd^
Body weight gain (g)	37.00 ± 5.10^bc^	32.33 ± 6.60^ac^	34.33 ± 4.50^bc^	21.00 ± 5.35^a^	41.00 ± 6.53^c^	25.67 ± 1.70^ab^
Growth rate per day (%)	0.47 ± 0.08^bc^	0.40 ± 0.10^ac^	0.50 ± 0.03^c^	0.31 ± 0.07^ab^	0.48 ± 0.09^c^	0.29 ± 0.03^a^
Food intake (g/day)	18.58 ± 0.16^a^	20.96 ± 4.53^a^	21.55 ± 3.67^a^	16.32 ± 2.23^a^	20.18 ± 2.34^a^	30.46 ± 1.16^b^
Food efficiency ratio (%)	7.12 ± 1.04^cd^	5.63 ± 1.45^bc^	5.74 ± 0.70^bd^	4.61 ± 1.10^ab^	7.26 ± 0.89^cd^	3.02 ± 0.32^a^
Female rats						
Initial body weight (g)	212.67 ± 22.07^a^	221.00 ± 20.40^a^	226.67 ± 5.79^a^	214.67 ± 10.87^a^	231.00 ± 4.55^a^	224.33 ± 8.18^a^
Final body weight (g)	225.00 ± 25.15^a^	233.67 ± 17.25^a^	236.67 ± 5.19^a^	230.00 ± 7.79^a^	244.33 ± 4.92^a^	235.67 ± 7.13^a^
Body weight gain (g)	12.33 ± 7.71^a^	12.67 ± 4.50^a^	10.00 ± 3.74^a^	15.33 ± 5.56^a^	13.33 ± 3.68^a^	11.33 ± 1.25^a^
Growth rate per day (%)	0.21 ± 0.13^a^	0.21 ± 0.08^a^	0.16 ± 0.06^a^	0.26 ± 0.10^a^	0.21 ± 0.06^a^	0.18 ± 0.03^a^
Food intake (g/day)	14.50 ± 2.89^a^	14.02 ± 0.66^a^	15.00 ± 1.88^a^	14.63 ± 0.49^a^	13.46 ± 0.75^a^	23.25 ± 1.61^b^
Food efficiency ratio (%)	2.86 ± 1.27^a^	3.25 ± 1.26^a^	2.48 ± 1.13^a^	3.76 ± 1.43^a^	3.51 ± 0.78^a^	1.74 ± 0.15^a^

Note

Results expressed as mean ± standard deviation (*SD*). Values not sharing a common superscript within a row are statistically significant (*p* < .05).

Abbreviations: NC, negative control; OL, olive oil; PC, positive control; PO, Passiflora oil.

### 3.5 Effect of *Passiflora edulis* on blood lipid profile

As shown in Table 5, rats fed with the diet enriched with lard showed deteriorated lipid profiles with significant increases in total cholesterol, VLDL, LDL‐cholesterol, triglycerides, and the atherogenic index, as compared with the normal control group (*p* < .05). However, no significant difference was found between the level of HDL‐cholesterol of animals fed with the high‐fat diet (PC) and that of those fed with the standard diet (NC) though it was lower in the PC group. It was found that passion fruit seed oil and olive oil supplementation significantly decreased the levels of triglycerides (*p* < .05) compared with the untreated group. Similar results were obtained with LDL‐cholesterol levels, which were significantly decreased in treated animals compared with the high‐fat diet group. As far as the HDL‐cholesterol is concerned, its level significantly increased in treated groups compared with the high‐fat diet group (*p* < .05) and the highest values were obtained with olive oil supplementation. However, there was no significant difference (*p* > .05). The atherogenic index was significantly decreased in the treated groups compared with the untreated ones. The lowest value was obtained with the group treated with *P. edulis* seed oil in both sexes. There was a negative correlation (*p* < .01) between HDL‐cholesterol levels and atherogenic index (*r* = −.49 and *r* = −.42 for males and females, respectively) and a positive correlation (*p* < .01) between LDL‐cholesterol levels and atherogenic index (*r* = .90 and *r* = .87 for males and females, respectively.

**Table 5:** Lipid profile of control and treatment groups in male and female rats

	PC	PO 1 ml	PO 2 ml	OL 1 ml	OL 2 ml	NC
Male (mg/dl)						
Triglyceride levels	96.47 ± 0.97^e^	90.7 ± 0.19^ce^	85.13 ± 1.48^cd^	84.15 ± 3.44^bc^	79.88 ± 5.56^abd^	76.25 ± 2.40^a^
Total cholesterol levels	147.234 ± 0.91^cd^	140.39 ± 2.47^b^	141.68 ± 0.04^b^	143.92 ± 4.21^bc^	144.05 ± 0.42^bd^	123.63 ± 0.65^a^
HDL‐cholesterol levels	34.47 ± 0.75^a^	41.3 ± 0.49^bd^	41.18 ± 0.25^bc^	39.83 ± 1.24^b^	41.89 ± 0.74^cd^	36.24 ± 1.31^a^
VLDL‐cholesterol levels	19.29 ± 0.19^e^	18.14 ± 0.04^cde^	17.03 ± 0.30^bd^	16.83 ± 0.69^bc^	15.98 ± 1.11^ab^	15.25 ± 0.48^a^
LDL‐cholesterol levels	93.47 ± 0.79^e^	80.95 ± 2.67^b^	83.47 ± 0.54^bc^	87.25 ± 4.87^cd^	86.18 ± 1.98^bd^	72.13 ± 1.48^a^
Atherogenic index	3.27 ± 0.15^e^	1.9 ± 0.14^b^	1.97 ± 0.02^bc^	2.22 ± 0.24^cd^	2.01 ± 0.09^bd^	1.46 ± 0.10^a^
Female (mg/dl)						
Triglyceride levels	86.53 ± 0.68^e^	80.04 ± 2.18^ab^	80.25 ± 2.12^ac^	76.96 ± 1.24^a^	80.47 ± 1.40^ad^	82.36 ± 2.78b^cde^
Total cholesterol levels)	150.21 ± 0.08^d^	143.13 ± 1.41^c^	132.34 ± 1.89^b^	149.79 ± 1.43^d^	149.71 ± 1.05^d^	124.93 ± 1.74^a^
HDL‐cholesterol levels	34.75 ± 2.56^a^	38.89 ± 1.50^b^	39.61 ± 0.6^b^	40.95 ± 0.02^bc^	42.96 ± 0.47^c^	35.85 ± 0.35^a^
VLDL‐cholesterol levels	17.31 ± 0.14^e^	16.01 ± 0.44^ab^	16.05 ± 0.42^ac^	15.39 ± 0.25^a^	16.09 ± 0.28^ad^	16.47 ± 0.56^bcde^
LDL‐cholesterol levels	98.15 ± 2.50^d^	88.24 ± 2.56^b^	76.67 ± 2.13^a^	93.45 ± 1.36^c^	90.65 ± 0.61^bc^	72.61 ± 2.35^a^
Atherogenic index	3.48 ± 0.62^c^	2.30 ± 0.25^b^	1.65 ± 0.13^a^	2.38 ± 0.07^b^	2.17 ± 0.02^ba^	1.57 ± 0.13^a^

Note

Results expressed as mean ± standard deviation (*SD*). Values not sharing a common superscript within a row are statistically significant.

Abbreviations: NC, negative control; OL, olive oil; PC, positive control; PO, Passiflora oil.

## 4 DISCUSSION

All fruits are generally regarded as rich in some nutrients such as vitamins and carbohydrates; however, little are good sources of lipid. In this study, the extraction yield of *P. edulis* seed oil was 19.90%. This value was slightly higher than that reported by Nyanzi et al. (2005), who have obtained a value of 18% with the same part of the same fruit in Uganda. This slight variation might be attributed to the agro‐climatic factors under which the plant was subjected. In fact, Gigon and Le Jeune (2010) state that oil content and different constituents of the oil vary according to the growing areas, the local agronomic practices, the variety, and the maturation stage of the fruits. Although *P. edulis* seeds are a good source of vegetable oil which has interesting physical properties (yellow gold and very light color, liquid at room temperature), one of the main qualitative aspects of oil is generally attached to its composition as well as its oxidative stability.

The quality and digestibility of edible vegetable oils are determined by the amount and composition of unsaturated fatty acids. Linoleic, oleic, palmitic, and stearic acids were the abundant fatty acids detected in the oil. The oil contained the essential fatty acids, which are linoleic acid (68.39%) and linolenic acid (0.54%). This fatty acid composition is in agreement with that obtained by Piombo et al. (2006). The fatty acid composition of Passiflora oil permit its classify among the other unsaturated oils, which are corn oil, grape seed oil, sunflower oil, soybean oil… (Codex Alimentarius, 2009). The obtained fatty acid profile, which is rich in unsaturated fatty acids and poor in saturated fatty acids, is considered to be ideal for consumption as such oils have been proven to have beneficial effects in humans and animals. However, it cannot be used for frying, but will be very good for seasoning like the other polyunsaturated oils. It can also be used in the formulation of other foods such as margarine. The presence of linoleic acid at appropriate levels is crucial, since it is an essential fatty acid. The comparison of the ratio saturated fatty acids/unsaturated fatty acids to those reported by Borges, Maia, Gomes, and Cavalcanti (2007) for common oils such as peanut, corn, and soybeans was found to be similar (1/5.64 for Passiflora oil and 1/6.70 for corn oil). Dubois, Breton, Linder, Fanni, and Parmentier (2007) ranked vegetable oils according to their fatty acid composition. Based on this classification, the passion fruit seed oil belongs to the class of polyunsaturated oils.

The degree of unsaturation of oil, expressed as its iodine value, can also serve as an indicator for its suitable use. When the iodine value is high, it means that the oil is rich in unsaturated fatty acids and can easily oxidize. In such circumstances, it should not be processed at high temperature. However, when the iodine value is low, the oil is rich in saturated fatty acids and then resistant toward oxidation reactions. Such oils are suitable for frying and soap and candle production. The iodine value of passion fruit seed oil was 97.4 ± 0.45 g I_2_/100 g, which was significantly higher, but lower than the 133–141 reported by Nyanzi et al. (2005) in the *Passiflora* seed oils from Uganda. A decrease in the iodine value is generally attributed to the destruction of the double bonds of its fatty acids by oxidation, scission, or polymerization (Tynek, Hazuka, Pawlowicz, & Dudek, 2001). In addition, oils generally contain other compounds capable of promoting oxidation; this includes free fatty acids, metals, and chlorophyll (Choe, 2008). The good quality of passion fruit seed oil can be verified by its low peroxide value, which is related to the development of oxidative reactions. The passion fruit seed oil presented a peroxide value of 2.09 ± 0.20 meq O_2_/kg, which fall in the accepted range of 0–15 meq O_2_/kg as recommended by the Codex Alimentarius (2009) for crude oils.

This study was designed to evaluate the effect of the west Cameroonian *P. edulis* variety seed oil, in relation to its composition, on the lipid profile of high‐fat diet–fed rats and in comparison with that of olive oil, whose beneficial effect on lipid profile and on health is well documented. It has been shown that beneficial effect of Mediterranean diet, well known as a diet with high consumption of olive oil and minimal amount of saturated fatty acids effective in controlling blood lipid levels and decreasing serum cholesterol, thus preventing atherosclerosis and other diseases (Mukuddem‐Petersen, Oosthuizen, & Jerling, 2005). The follow‐up of the daily food intake during the experimentation indicates that the male and female rats consuming the lard had exhibited the lowest food intake. This high‐fat diet might have lowered their appetite. Indeed, this food was rich in saturated fatty acids and therefore was difficult to digest. The administration of different doses of *P. edulis* seed oil and olive oil positively improves food intake in males. These observations are in agreement with those of Pan and Storlien (1993), who noted that rats consuming a high‐fat diet had a lower food intake than those consuming a diet supplemented with olive oil. Moreover, animals under *P. edulis* seed oil treatment tend to have more appetite. However, the effect of the oil dose is not noticeable. Food intake appears to be related to weight gain in induced and untreated rats. The change in body weight is low in the male and female rats consuming a high‐fat diet. This indicates that this diet is of low nutritional quality and may contain elements that are not healthy and affect the growth of rats. However, the rats treated with the different doses of oils show an improvement in the growth rate, which indicates that these oils could be of good nutritional quality. The rats treated with olive oil showed a change in weight greater than that in rats treated with *P. edulis* seed oil. These results are in agreement with those of Pan and Storlien (1993), who showed that olive oil improved the body weight of rats.

High‐fat diet is widely used in the experimental induction of weight gain and obesity in animals, and it has been found that obesity is associated with the development of dyslipidemia and coronary heart diseases (CHDs)**.** The major risk factor is the lifestyle: lack of physical training, smoking, excessive food intake and wrong dietary pattern leading to overweight or obesity, high blood pressure, and high level of total cholesterol, LDL‐cholesterol, and triglycerides (Elmadfa & Kornsteiner, 2009). In this study, the levels of serum total cholesterol, triglycerides, and LDL‐cholesterol were in general significantly lower in the groups treated with *P. edulis* and olive oils compared with the high‐fat diet group. However, the level of HDL‐cholesterol was significantly high with *P. edulis* oil, demonstrating that dietary supplementation with this oil improves lipid profiles in rats. Indeed, strong evidence exists from clinical and intervention studies that the lipids in the diet are of a series of diseases or pathological conditions known as “diseases of civilization” (Mangas‐Cruz et al., 2004). The role of dietary fats in cardiovascular disease (CVD) and many other disorders has been often discussed with general conclusions that this process is not simple, but rather the consequence of complex factors. Nevertheless, each group of fatty acids has a specific role in many biologic pathways. The literature suggests that higher intakes of omega‐6 (or n‐6) polyunsaturated fatty acids (PUFAs) reduce the risk for coronary heart disease (CHD; Harris et al., 2009; Kermanshahi Pour, MacEachern, & Mirmehrabi, 2017). Linoleic acid is one of the most important PUFAs in human food because of its cardiovascular preventing effect. Linoleic acid reduces serum cholesterol and LDL level (FAO, 1994). The presence of high amounts of linoleic acid in the oil of this fruit suggests that PE seeds are highly nutritious and hence could be used in the management of hyperlipidemia. In fact, linoleic (18:2, n‐6) and alpha‐linolenic acids (18:3, n‐3) cannot be synthesized by humans and are regarded as essential fatty acids. Sufficient dietary intake of n‐3 or n‐6 fatty acids is crucial to achieve nutrient adequacy and to prevent and treat chronic diseases (Simopoulos, 1999).

Differences and variations observed between males and females can be attributed to hormonal variations generally observed in the female sex. Oestradiols, for example, significantly modify the lipid profile toward an anti‐atheromatous profile. It particularly decreases the total cholesterol by increasing the HDL/LDL ratio. It also decreases plasma triglyceride concentrations and increases LDL resistance toward oxidation. This is one of the reasons why men are highly exposed to cardiovascular diseases compared with premenopausal women (Manassier, 2013).

## 5 CONCLUSION

*Passiflora edulis* seeds from west Cameroon are a good source of vegetable oil with good proportion of omega‐6 fatty acids, which could positively affect the lipid profile of rats. Administration of *P. edulis* seed oil to rats reduces the triglyceride, total cholesterol, and LDL‐cholesterol levels and increases HDL‐cholesterol levels in Wistar rats. Its activity was comparable to that of olive oil.

## CONFLICT OF INTEREST

The authors declare that they have no conflict of interest.

## ETHICAL APPROVAL

This study involved animal testing and was approved by the Institutional Review Board of the University of Dschang, Cameroon. The protocol and procedures employed were ethically reviewed and approved by the same organ.
